# Prognostic Impact of Klintrup–Mäkinen (KM) Score in Gastric Cancer and Its Association with Pathological Parameters

**DOI:** 10.3390/medicina61040715

**Published:** 2025-04-13

**Authors:** Andreea-Raluca Cozac-Szőke, Georgian-Nicolae Radu, Anca Negovan, Dan Alexandru Cozac, Sabin Turdean, Andreea-Cătălina Tinca, Emőke-Andrea Szász, Iuliu-Gabriel Cocuz, Adrian-Horațiu Sabău, Raluca Niculescu, Diana Maria Chiorean, Alexandru Nicușor Tomuț, Ovidiu Simion Cotoi

**Affiliations:** 1 Doctoral School of Medicine and Pharmacy, George Emil Palade University of Medicine, Pharmacy, Science and Technology of Targu Mures, 540142 Targu Mures, Romania; andreea-raluca.szoke@umfst.ro (A.-R.C.-S.); dan-alexandru.cozac@umfst.ro (D.A.C.); adrian-horatiu.sabau@umfst.ro (A.-H.S.); raluca.niculescu@umfst.ro (R.N.); diana.chiorean@umfst.ro (D.M.C.); 2 Pathophysiology Department, George Emil Palade University of Medicine, Pharmacy, Science, and Technology of Targu Mures, 540142 Targu Mures, Romania; andreea-catalina.tinca@umfst.ro (A.-C.T.); iuliu.cocuz@umfst.ro (I.-G.C.); ovidiu.cotoi@umfst.ro (O.S.C.); 3 Faculty of Medicine, George Emil Palade University of Medicine, Pharmacy, Science, and Technology of Targu Mures, 540142 Targu Mures, Romania; nicusortomut19@gmail.com; 4 Department of Clinical Science-Internal Medicine, George Emil Palade University of Medicine, Pharmacy, Science, and Technology of Targu Mures, 540142 Targu Mures, Romania; ancanegovan@yahoo.com; 5 Department of Pathology, George Emil Palade University of Medicine, Pharmacy, Science and Technology of Targu Mures, 540142 Targu Mures, Romania; sabin.turdean@umfst.ro; 6 Department of Histology, George Emil Palade University of Medicine, Pharmacy, Science, and Technology of Targu Mures, 540142 Targu Mures, Romania; emoke.szasz@umfst.ro; 7 Pathology Department, Mures Clinical County Hospital, 540011 Targu Mures, Romania

**Keywords:** Klintrup–Mäkinen score, gastric cancer, prognosis, inflammation, histology

## Abstract

*Background and Objectives:* Gastric cancer (GC) remains a significant global health challenge with a poor prognosis. This study aimed to evaluate the association between Klintrup–Mäkinen (KM) inflammatory infiltrate grading and clinicopathological features in gastric cancer patients, investigating its potential as a prognostic marker. *Material and Methods:* This retrospective study analyzed 133 gastric adenocarcinoma patients diagnosed between 2020 and 2021 at County Clinical Hospital in Târgu Mureș, Romania. Patients were divided into two groups based on KM grades: low (grades 0–1, *n* = 62) and high (grades 2–3, *n* = 71). Clinicopathological characteristics and survival outcomes were compared between the groups. *Results:* Demographic characteristics were similar between the groups. Patients with low KM grades demonstrated significantly more aggressive tumor features, including a higher prevalence of Borrmann classification types III-IV (75.8% vs. 54.9%, *p* = 0.01), poorly differentiated histology (74.1% vs. 33.8%, *p* < 0.0001), advanced T stage (93.5% vs. 80.2%, *p* = 0.04), and lymph node involvement (87% vs. 60.5%, *p* = 0.0008). This group also exhibited higher rates of lymphatic invasion (79% vs. 50.7%, *p* = 0.001), venous invasion (51.6% vs. 30.9%, *p* = 0.02), perineural invasion (50% vs. 22.5%, *p* = 0.001), and positive surgical margins (32.2% vs. 15.4%, *p* = 0.02). Survival analysis revealed a hazard ratio of 1.642 (95% CI: 1.02–2.62) for patients with low KM grades compared to those with high KM grades. *Conclusions:* Low KM grades are associated with more aggressive tumor characteristics and poorer prognosis in GC patients. The KM score may serve as a valuable, cost-effective histological marker for assessing tumor aggressiveness and could aid in risk stratification when applied to routine H&E-stained slides. While it does not replace immunohistochemical or molecular analyses, integrating the KM score into pathological assessment may enhance prognostic accuracy and support identifying patients who might benefit from immunotherapy.

## 1. Introduction

Gastric cancer (GC) is the fifth most common cancer and a leading cause of mortality worldwide [1,2]. It can be classified into two anatomical sites: cardia GC (the upper part near the gastroesophageal junction) and non-cardia GC (the lower part of the stomach) [3]. Each of these has a distinct epidemiology and risk factors. GC has two main histological types: intestinal type (where tumor cells form glandular or tubular structures resembling the intestinal epithelium) and diffuse (noted for the absence of gland formation and poorly cohesive cells, including signet-ring cells, which exhibit a diffuse infiltrative growth pattern) [4].

Tumor–node–metastasis (TNM) staging and histological classification are standard predictive factors but essential in assessing patients with GC. Nonetheless, the latest research indicates that the tumor microenvironment (TME), particularly the immune response, is a crucial variable in determining tumor progression, the efficacy of immunotherapy, and the patient’s overall survival [5]. Known as “hot tumors”, cancers with significant immune cell infiltration typically react better to immunotherapy (checkpoint inhibitors targeting PD-1/PD-L1). On the other hand, “cold tumors” with low levels of immune infiltration frequently exhibit immune evasion strategies and react poorly to immune-based therapies [6,7].

Another important factor in prognosis is the location of immune infiltrates. Although both peritumoral inflammatory infiltrates (at the invasive margin) and intratumoral lymphocytes (within tumor nests) support the host immune response, their prognostic relevance can differ. Peritumoral immune cells, especially at the invasive front [8], reflect the dynamic interaction between the tumor and the immune system and are critical for predicting disease progression.

In addition to these histological considerations, certain molecular features of GC have been linked to the extent of tumor immune infiltration and patient prognosis. For example, microsatellite instability-high (MSI-H) tumors—arising from deficient mismatch repair (dMMR)—typically harbor a high mutational burden that generates abundant neoantigens, leading to pronounced lymphocytic infiltration [9]. Clinically, MSI-H/dMMR GC represents roughly 8–10% of cases and has been associated with earlier-stage disease and improved survival compared to microsatellite-stable tumors [9]. Likewise, Epstein–Barr virus-positive gastric cancers (EBV-positive GCs, ~5–10% of cases) are characterized by an “immune-hot” microenvironment with dense T-cell infiltrates, which is rarely seen in EBV-negative tumors. Notably, EBV-associated GCs tend to show slightly better outcomes and distinct patterns of immune checkpoint expression [10,11].

These molecular subtypes underscore the connection between tumor genomics and the local immune response. Tumors with MSI-H/dMMR or EBV infection often elicit robust immune cell recruitment to the invasive margin, a feature directly evaluated by the Klintrup–Mäkinen (KM) score [12,13].

The KM score is a standard histopathological grading system used to assess the level of inflammatory cell infiltration at the tumor’s invasive margin on routine hematoxylin and eosin (H&E) staining [14]. The KM score was first developed for colorectal cancer, but it is currently being investigated for other cancer types [15], including GC, to determine whether it is a valid prognostic indicator [16,17].

An immune-rich molecular subtype of GC would be expected to have a high KM grade on histology, reinforcing the prognostic significance of immune infiltration in this disease. This understanding provides context for immune-based scoring systems like the KM grade, which aim to capture the host–tumor immune interaction in routine pathology.

This study aims to evaluate the applicability of the KM score in routine pathology for assessing inflammatory infiltrates in GC and to examine its prognostic significance in conjunction with established factors (such as tumor stage, histological grade, lymph node involvement, etc.) in the management of GC.

## 2. Materials and Method

### 2.1. Study Design and Case Selection

This retrospective study included 133 patients diagnosed with gastric adenocarcinoma between 2020 and 2021 at the County Clinical Hospital Pathology Department in Târgu Mureș, Romania. Inclusion criteria were as follows: (1) histopathological diagnosis of primary gastric adenocarcinoma; (2) no previous neoadjuvant chemotherapy or radiotherapy; (3) availability of at least one slide containing the tumor’s invasive margin; and (4) complete medical records along with follow-up data. Informed consent was obtained from all the patients involved in this study. This study was conducted following the Declaration of Helsinki and was approved by the County Clinical Hospital Ethics Committee in Târgu Mureș, Romania, protocol number 13106/09.09.2024.

Clinical data, including age, sex, and survival status, were reviewed from patient records and pathology reports. Pathological data were also assessed, including tumor location, Borrmann type, Lauren classification, tumor size, grade, pT category, lymphatic invasion, venous invasion, perineural invasion, lymph node status, distant metastasis, and resection margin status. Histological staging (well/moderately/poorly differentiated) and TNM staging were determined according to the 9th edition of the American Joint Committee on Cancer (AJCC) Staging Manual.

From an initial cohort of 156 patients, 133 were selected for inclusion in the study; the remaining 23 patients did not satisfy the inclusion criteria and were excluded from the analysis. Of the 133 included patients, complete three-year follow-up data were available for 129 patients, while 4 patients had incomplete follow-up information.

Further, we divided patients into two groups based on KM grades [18]. Patients with grades 0–1 were categorized as the “Patients with low KM grades group” (*n* = 62), while those with grades 2–3 were classified as the “Patients with high KM grades group” (*n* = 71).

### 2.2. Pathohistological Assessment

H&E-stained slides, retrospectively collected and previously used for clinical decision-making, were retrieved for analysis under Zeiss Axio Lab A1 Light Microscope. All H&E slides were re-examined to identify at least one representative slide showing the tumor’s invasive margin. This retrospective analysis ensured the accurate evaluation of the tumor’s depth and invasive characteristics.

Two pathologists blinded to the data independently assessed the KM grade by reviewing the slides. KM grade was evaluated based on the inflammatory cell infiltrate at the invasive edge of the tumor. Each case was assigned a grade from 0 to 3, as follows: Grade 0: no increase in inflammatory cells; Grade 1: mild, patchy clusters of inflammatory cells; Grade 2: a band-like inflammatory infiltrate at the tumor’s invasive margin with some evidence of cancer cell destruction; and Grade 3: prominent inflammatory reaction accompanied by a destruction of cancer cells [18].

**Figure 1:**
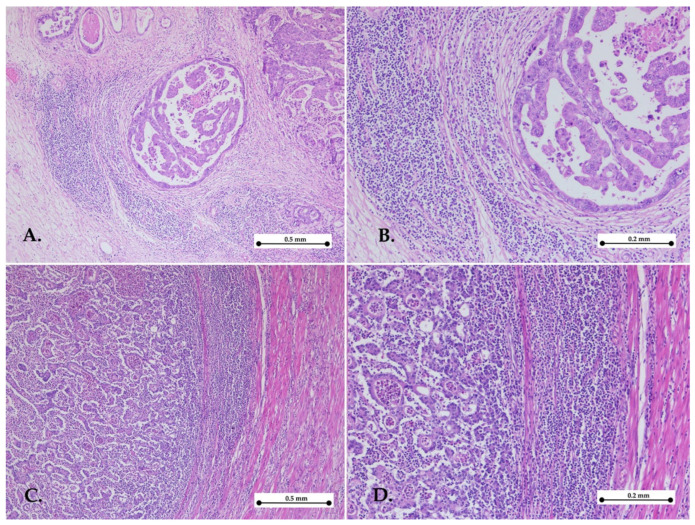
Representative images of high Klintrup–Mäkinen grade. Gastric cancer with high KM grade showing abundant cup-like inflammatory infiltrates in the invasive front—hematoxylin–eosin stain, ×5 objective (**A**,**C**); objective ×10 (**B**,**D**).

**Figure 2:**
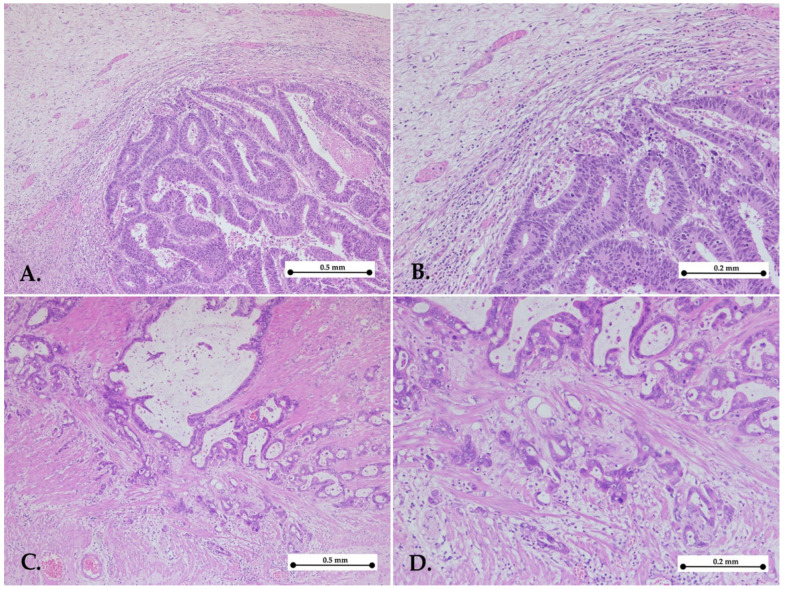
Representative images of low Klintrup–Mäkinen grade. Gastric cancers with low KM grade showing mild and patchy inflammation (**A**,**B**) or no increase in inflammatory cells (**C**,**D**) in the invasive edge of the tumor—hematoxylin–eosin stain, ×5 objective (**A**,**C**); ×10 objective (**B**,**D**).

For the statistical analysis, grades 0–1 were categorized as low KM grades, while grades 2–3 were classified as high KM grades. In cases where one pathologist scored a low KM grade and the other a high KM grade, a third pathologist was consulted, and the slides were reassessed (Figure 1 and Figure 2).

The presence of *Helicobacter pylori* (*H. pylori*) infection was confirmed on H&E and Giemsa stains.

Active inflammation was histologically characterized by the presence of neutrophils, while chronic inflammation was considered when the inflammatory infiltrate was composed of lymphocytes, plasma cells, and macrophages in the gastric mucosal and submucosal layers.

Glandular atrophy is defined by the loss or reduction in the number and size of gastric glands, resulting in the thinning of the mucosa.

Complete intestinal metaplasia was identified when the normal gastric mucosal epithelium was replaced by an intestinal-type epithelium, consisting of absorptive enterocytes and mucus-secreting goblet cells. Incomplete intestinal metaplasia was characterized by the presence of goblet cells but without full differentiation into enterocytes.

The Borrmann classification was used to categorize GC based on its macroscopic appearance. It divided the GC into four distinct types: Type I—polypoid; Type II—fungating; Type III—ulcerating; and Type IV—Infiltrative.

### 2.3. Statistical Analysis

Statistical analysis was performed using GraphPad Prism, version 10.4.1. Continuous variables are presented as the median and interquartile range (IQR), while qualitative data are expressed using frequencies and percentages. Differences between the ages of the two study groups were analyzed using Fisher’s exact test for categorical data and the Mann–Whitney test for continuous variables. The significance level was set at α = 0.05, based on the study population size and international consensus. All tests were two-sided, with a *p*-value of <0.05 considered statistically significant.

## 3. Results

The median age was comparable between the two groups. Patients in the low-KM-grade group had a median age of 71 years (IQR: 59.0–76.0), while those in the high-KM-grade group had a median age of 68 years (IQR: 60.5–73.5). The Mann–Whitney U test revealed no statistically significant difference between the groups (*p* = 0.53).

Analysis of clinicopathological characteristics between low KM grades (*n* = 62) and high KM grades (*n* = 71) groups revealed similar demographic distributions, with no significant differences in gender (*p* = 0.55) or age (*p* = 0.85).

Significant differences were observed in several key pathological parameters. Patients in the low KM group had a significantly higher proportion of advanced tumors classified as Borrmann III and IV (75.8% vs. 54.9%, *p* = 0.01) with an OR of 2.57 (95% CI: 1.20–5.26). Histologically, this group was strongly associated with poorly differentiated tumors (G3: 74.1% vs. 33.8%, *p* < 0.0001), showing a notable OR of 5.6 (95% CI: 2.63–11.39). Depth-of-invasion analysis revealed a higher prevalence of advanced pT stage (pT3–4) in patients in the low-KM-grade group (93.5% vs. 80.2%, *p* = 0.04), with an OR of 3.5 (95% CI: 1.11–10.33) (Table 1).

Additionally, lymph node involvement was significantly more frequent in this group (pN1–3: 87% vs. 60.5%, *p* = 0.0008), with an OR of 4.3 (95% CI: 1.80–10.19). Regarding invasion patterns, patients with low KM grades exhibited significantly higher rates of lymphatic invasion (79% vs. 50.7%, *p* = 0.001, OR 3.66, 95% CI: 1.69–7.82), venous invasion (51.6% vs. 30.9%, *p* = 0.02, OR 2.37, 95% CI: 1.18–4.65), and perineural invasion (50% vs. 22.5%, *p* = 0.001, OR 3.4, 95% CI: 1.60–7.18). Positive surgical margins were also more frequently observed in this group (32.2% vs. 15.4%, *p* = 0.02), with an OR of 2.59 (95% CI: 1.09–5.74). However, no significant differences were found between the groups regarding tumor size (*p* = 0.48) or the presence of distant metastases (*p* = 0.49) (Table 1).

No significant differences were found between the groups in terms of *H. pylori* infection status, inflammatory patterns, glandular atrophy, or complete and incomplete intestinal metaplasia. All parameters analyzed did not reach statistical significance (*p* > 0.05) (Table 2).

The survival analysis for the two groups revealed significant findings when using the log-rank (Mantel–Cox) test, with a chi-square value of 4.32 and a *p*-value of 0.03 (Figure 1).

**Figure 3:**
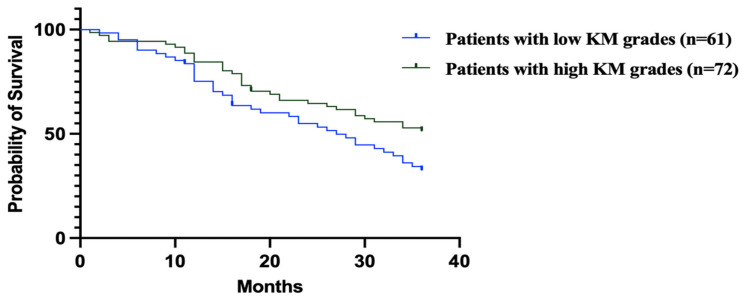
Mantel–Haenszel analysis revealed patients with low KM grades had significantly worse survival (HR = 1.642, 95% CI: 1.02–2.62) compared to those with high KM grades. Conversely, high KM grades were associated with improved survival (HR = 0.6, 95% CI: 0.38–0.97).

The hazard ratio (HR) from the Mantel–Haenszel test shows a ratio of 1.642 for patients with low KM grades compared to high KM grades, with a 95% CI ranging from 1.02 to 2.62. Conversely, the reciprocal HR for high KM grades compared to low grades is 0.6, with a 95% CI of 0.38 to 0.97 (Figure 3).

Figure 3 shows the Kaplan–Meier survival curves comparing overall survival in GC patients with low vs. high KM grades.

**Figure 4:**
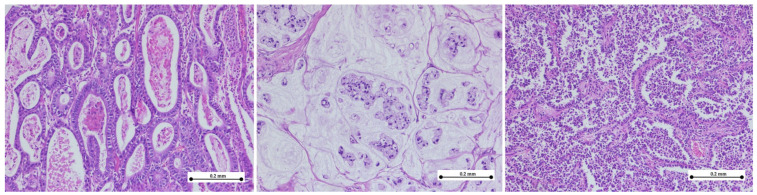
Histologic subtypes of gastric adenocarcinoma. (**Left**) Tubular adenocarcinoma composed of neoplastic ducts of varying size and shape; (**middle**) mucinous adenocarcinoma exhibiting extracellular mucin pools with nests of signet-ring cells; (**right**) tubulo-papillary adenocarcinoma showing finger-like projections composed of neoplastic cells arranged around fibrovascular cores. (hematoxylin–eosin stain, ×10).

In the histopathological analysis of gastric adenocarcinoma specimens, we identified distinct morphological subtypes, as can be seen in Figure 4.

## 4. Discussion

While the KM score is not established as a prognostic marker in GC, it has gained increasing attention in recent years due to its potential role in understanding the immune landscape of tumors [8,18]. This research study showed that the KM score could serve as a valuable prognostic marker in GC, as patients with higher KM scores, which reflect a stronger immune response at the tumor site, had better prognoses and improved survival outcomes. In contrast, lower KM scores, indicative of a weaker immune response, have been linked to poorer prognosis, more aggressive tumor behavior, and a higher likelihood of metastasis. These observations suggest that the intensity of the local immune infiltrate (captured by KM grading) is an important correlate of tumor aggressiveness in GC.

Although the KM score has been primarily studied in GC and colorectal cancer, its underlying principle—the evaluation of immune cell infiltration at the invasive tumor margin—has broader relevance. In other malignancies, such as pancreatic, esophageal, and lung cancer, various studies have explored similar peritumoral immune patterns, albeit using different evaluation systems. These findings suggest that a KM-like approach could be adapted and validated in additional tumor types, even if the KM score has not yet been studied in cancers outside the gastrointestinal tract. In colorectal cancer—the disease setting in which the KM grading was first developed—a high KM infiltrate grade is well known to predict favorable outcomes [13,18]. Early investigations applying KM scoring to GC were limited in size, but they similarly noted trends toward improved survival with pronounced inflammatory cell infiltration at the tumor margin. More recently, a large study of 741 GC patients by Kemi et al. (2020) demonstrated that a high KM grade was an independent predictor of longer 5-year survival, with an adjusted HR of ~0.59 for mortality in high- vs. low-KM groups [18]. In that study, KM grading even outperformed an immunohistochemistry-based immune cell score in prognostic value, reinforcing that the KM score captures clinically relevant immune involvement in GC [18]. Our current results are in line with these reports, underscoring that a strong immune infiltrate at the invasive front (high KM score) is associated with less aggressive disease and better patient outcomes.

Although our study did not directly evaluate MSI, dMMR, or EBV status, these molecular factors likely intersect with the prognostic value of KM scoring. MSI-H and EBV-positive GCs are known to exhibit intense immune responses in the TME, characterized by abundant tumor-infiltrating lymphocytes [10,11,13]. Theoretical considerations suggest that cases with these immunogenic features would tend to receive high KM grades due to their florid inflammatory infiltrates. This could partly explain the improved outcomes typically observed in MSI-H/dMMR and EBV-positive GCs [9,10]. Conversely, tumors lacking such immunogenic stimuli (e.g., microsatellite-stable, EBV-negative cancers) often exhibit a “cold” TME with sparse immune cells [10,13], aligning with low KM scores and a more aggressive clinical behavior. In essence, the KM score may be acting as a surrogate marker for an immunologically active tumor subtype. Integrating molecular profiling in future KM studies would be valuable to confirm this interplay—for instance, in determining whether high-KM tumors in a cohort are enriched for dMMR/MSI or EBV infection. Such information would strengthen the biological rationale for why KM-high patients have better survival, potentially linking it to well-established favorable molecular subgroups.

Looking ahead, further characterization of the TME in GC should accompany KM scoring to deepen our understanding and improve patient stratification. Advanced techniques like multiplex immunohistochemistry (mIHC) allow for the simultaneous visualization of multiple immune cell markers within the tumor tissue, providing insights into the composition and spatial distribution of the immune infiltrate [19]. Recent studies have applied mIHC and multispectral imaging to GC, profiling dozens of immune cell subsets and their functional states in situ [20]. These approaches can reveal, for example, the balance of cytotoxic T cells vs. regulatory T cells or the polarization of tumor-associated macrophages within the invasive margin. Spatial immune profiling—including digital image analysis of immune cell localization—has already shown prognostic value in other gastrointestinal cancers by capturing not just the density but the organization of immune cells relative to tumor cells [21].

In the present study, the analysis of clinicopathological characteristics between low and high KM grades revealed several important findings that contribute to our understanding of the relationship between KM grades and the aggressiveness of GC.

While demographic factors such as gender, age, and type of gastrectomy did not show significant differences between the two groups, several pathological features were associated with high KM grades, suggesting that KM grading may serve as an important prognostic indicator in GC.

One of the most significant findings was the higher proportion of advanced tumors in the low KM grade group, with a notably higher frequency of Borrmann III and IV classifications. These types of tumors are generally associated with more aggressive disease behavior and a poorer prognosis [22,23]. Furthermore, histological analysis revealed that low KM grades were strongly associated with poorly differentiated tumors (G3). These tumors have a higher tendency for invasion and metastasis [24], which may explain the observed poorer survival outcomes in these patients.

The analysis showed that patients with low KM grades had significantly more advanced pT stages (pT3–4), indicating deeper tumor invasion. This was associated with a higher risk of lymph node metastasis, which was more frequent in the low-KM-grade group (OR 4.3), further supporting the link between low KM grades and more aggressive tumor biology [25].

Likewise, the association of low KM grades with higher rates of lymphatic, venous, and perineural invasion suggests that the KM score may serve as an indicator of aggressive tumor behavior. Additionally, the significantly higher frequency of positive surgical margins in this group indicates that tumors with low KM scores often have an infiltrative growth pattern with poorly defined boundaries, making complete surgical resection more challenging.

Interestingly, no significant differences were observed between the two groups concerning tumor size, distant metastasis, or *H. pylori* infection status. This is significant because, while *H. pylori* infection is a well-established risk factor for GC [26,27], its presence did not correlate with KM grades in the studied population.

Furthermore, the similar proportions of inflammatory patterns, glandular atrophy, and intestinal metaplasia between the two groups suggest that these factors may not be as closely related to KM grade, highlighting the possibility that KM grade is more reflective of the biological behavior of the tumor rather than associated inflammatory or metaplastic changes.

### Future Perspectives and Study Limitations

We propose that future studies of KM scoring should incorporate multidimensional immune analyses. Multiplex IHC and spatial transcriptomic or advanced imaging can help identify which specific immune cell subsets (CD8+ T cells, FoxP3+ Tregs, CD68+ macrophages, etc.) are most associated with high KM scores and how these cells are distributed within the TME. This level of detail could enhance the biological interpretation of KM grades and further validate their prognostic value.

Combining KM histological assessment with in-depth immune profiling and molecular features like MSI and EBV status could yield a more comprehensive prognostic model. Importantly, such integration may also inform therapeutic strategies, as tumors with high KM scores and immune-rich environments are likely to be more responsive to immunotherapy or other immune-modulating treatments.

While the KM score does not replace immunohistochemistry or molecular analysis in characterizing the immune landscape, it offers a practical and cost-effective alternative for routine evaluation because it can be performed on routine H&E-stained sections. Its integration into GC assessment could enhance risk stratification [21], complement existing prognostic factors, and potentially guide treatment decisions, particularly in the context of immunotherapy [28,29].

However, because the KM score is still being evaluated in clinical studies [30,31,32], its widespread use in histopathological reports is not yet universal. Incorporating the KM score into routine reports would require standardized protocols for its evaluation and interpretation. Additionally, pathologists would need specific training to assess and report the score in a consistent and reproducible manner.

Some limitations of our study should be acknowledged. This was a single-center study with a moderate sample size and a limited timeframe. We did not incorporate molecular profiling (MMR status, MSI testing, or EBV testing) into our analysis, so we could not directly evaluate how much of the prognostic impact of KM grade might be attributable to underlying molecular subtypes. We have highlighted this as a priority for future research. Additionally, our immune evaluation relied solely on H&E staining, without immunohistochemical or molecular markers. Despite these limitations, our findings support the growing evidence that KM scoring has prognostic value in GC.

## 5. Conclusions

Our findings highlight the association between low KM grades and more aggressive tumor characteristics in GC, including deeper invasion, increased lymph node involvement, and higher rates of lymphovascular and perineural invasion. While it does not replace immunohistochemical or molecular analyses, its application as part of standard pathology could enhance prognostic accuracy. Future studies integrating KM scoring with molecular markers such as MSI/dMMR and EBV status, as well as advanced immune profiling techniques, will help validate and refine the role of KM score in guiding GC patient management. Integrating the KM score into pathological assessment may ultimately support identifying patients who may benefit from immunotherapy or more aggressive treatment based on the immune context of their tumors, moving toward a more personalized management of GC.

## Figures and Tables

**Table 1:** Comparison of clinicopathological parameters between patients with low vs. high KM grades.

Variable	Patients with Low KM Grades *n* = 62		Patients with High KM Grades *n* = 71		*p*-Value *	OR	95% CI
	*n*	%	*n*	%			
Gender					0.55	1.3	0.61 to 2.73
Males	47	75.8	50	70.4			
Females	15	24.2	21	29.6			
Age					0.85	0.89	0.43 to 1.86
≥65 years	38	61.2	46	64.7			
<65 years	23	38.8	25	35.3			
Gastrectomy					0.85	1.1	0.53 to 2.31
Total	28	45.1	26	36.6			
Partial	34	54.9	45	63.4			
Borrmann clasification					0.01	2.57	1.20 to 5.26
III and IV	47	75.8	39	54.9			
I and II	15	24.2	32	45.1			
Size					0.48	1.32	0.68 to 2.60
≥5 cm	34	54.8	34	47.8			
<5 cm	28	45.2	37	52.2			
Histological grade							
G3 (poorly differentiated)	46	74.1	24	33.8	<0.0001	5.6	2.63 to 11.39
G1/G2 (well/moderately differentiated)	16	25.9	47	66.2			
Depth of invasion (pT stage)					0.04	3.5	1.11 to 10.33
pT3–4	58	93.5	57	80.2			
pT1–2	4	6,5	14	19.8			
Lymph node status (pN stage)					0.0008	4.3	1.80 to 10.19
pN1–3	54	87	43	60.5			
pN0	8	13	28	39.5			
Distant metastasis (pM stage)					0.49	1.4	0.61 to 3.4
pM1	13	21	11	15.4			
pM0	49	79	60	84.6			
Lymphatic invasion					0.001	3.66	1.69 to 7.82
Present	49	79	36	50.7			
Absent	13	21	35	49.3			
Venous invasion					0.02	2.37	1.18 to 4.65
Present	32	51.6	22	30.9			
Absent	30	48.4	49	69.1			
Perineural invasion					0.001	3.4	1.60 to 7.18
Present	31	50	16	22.5			
Absent	31	50	55	77.5			
Surgical margins					0.02	2.59	1.09 to 5.74
Positive	20	32.2	11	15.4			
Negative	42	67.8	60	84.6			

* Obtained from c hi-square or Fisher’s exact tests; OR: odds ratio; CI: 95% confidence interval.

**Table 2:** Histopathological features and *H. pylori* status between patients with low vs. high KM grades.

Variable	Patients with Low KM Grades *n* = 62		Patients with High KM Grades *n* = 71		*p*-Value *	OR	95% CI
	*n*	%	*n*	%			
*H. pylori* status							
Positive	11	17.7	11	15.4	0.8	1.17	0.47 to 2.92
Negative	51	82.3	60	84.6			
Inflammation							
Active inflammation	24	38.7	32	45	0.48	0.76	0.38 to 1.52
Chronic inflammation	38	61.3	39	55			
Glandular atrophy							
Yes	40	64.5	45	63.4	>0.99	1.05	0.51 to 2.91
No	22	35.5	26	36.6			
Complete intestinal metaplasia							
Yes	33	53.2	37	52.1	>0.99	1.04	0.53 to 2.04
No	29	46.8	34	47.9			
Incomplete intestinal metaplasia							
Yes	36	58	48	67.6	0.2	0.66	0.31 to 1.35
No	26	42	23	32.4			

* Obtained from chi-square or Fisher’s exact tests; OR: odds ratio; CI: 95% confidence interval.

## Data Availability

The datasets used and analyzed during the current study are available from the corresponding author upon request.
